# Human Pluripotent Stem-Cell-Derived Models as a Missing Link in Drug Discovery and Development

**DOI:** 10.3390/ph14060525

**Published:** 2021-05-30

**Authors:** Xiying Lin, Jiayu Tang, Yan-Ru Lou

**Affiliations:** Department of Clinical Pharmacy and Drug Administration, School of Pharmacy, Fudan University, Shanghai 201203, China; 20211030094@fudan.edu.cn (X.L.); 15307130212@fudan.edu.cn (J.T.)

**Keywords:** human pluripotent stem cells, human embryonic stem cells, human-induced pluripotent stem cells, drug discovery, drug development

## Abstract

Human pluripotent stem cells (hPSCs), including human embryonic stem cells (hESCs) and human-induced pluripotent stem cells (hiPSCs), have the potential to accelerate the drug discovery and development process. In this review, by analyzing each stage of the drug discovery and development process, we identified the active role of hPSC-derived in vitro models in phenotypic screening, target-based screening, target validation, toxicology evaluation, precision medicine, clinical trial in a dish, and post-clinical studies. Patient-derived or genome-edited PSCs can generate valid in vitro models for dissecting disease mechanisms, discovering novel drug targets, screening drug candidates, and preclinically and post-clinically evaluating drug safety and efficacy. With the advances in modern biotechnologies and developmental biology, hPSC-derived in vitro models will hopefully improve the cost-effectiveness and the success rate of drug discovery and development.

## 1. Brief Introduction to Human Pluripotent Stem Cells (hPSCs) and Their Applications

Human pluripotent stem cells (hPSCs), including human embryonic stem cells (hESCs) and human induced pluripotent stem cells (hiPSCs), are cells with the ability to self-renew and to develop into all cell types in a human adult body [1]. hESCs come from the blastocyst, which is a developing embryo 5–6 days after fertilization. As for hiPSCs, they are derived from reprogrammed somatic cells with ectopic expression of pluripotency factors, like OSKM (OCT4/SOX2/KLF4/C-MYC) [2].

Grown in vitro, hPSCs show the potential to generate all lineages of the embryo in vivo and can differentiate into all types of somatic cells in vitro, becoming a popular and valuable cellular source for the treatment of many degenerative diseases, including Parkinson’s disease (PD), Alzheimer’s disease (AD), and age-related macular degeneration (AMD) [3], as well as injuries to tissues lacking regeneration capability such as ischemic heart failure [4], diabetes, and spinal cord injuries [5]. Moreover, hPSCs can be used for tissue and organ reconstruction in vitro. A typical approach is to culture isolated hPSCs on a supportive matrix to provide a three-dimensional (3D) growth environment for stem cells. At the same time, based on the understanding of organ development in vivo, specific cytokines and growth factors are added to the matrix to induce differentiation into specific organs. In such cultures, stem cells differentiate and self-assemble to form organ-like structures like organs or tissues in the body, which are called organoids. An organoid [6,7] is a 3D cell model constructed in vitro by stem cells or progenitors grown in extracellular matrix hydrogels or in biomaterial-free 3D conditions according to the physiological structure of the organ. It can mimic the cell type, structure, and function of the corresponding organ. Depending on the study, these cells can be derived from tissues of healthy and diseased people and from hPSCs. If organoids can be applied to organ transplantation, it will largely solve the problem of the current organ shortage and organ transplantation.

Besides their in vivo applications, hPSCs have the potential to accelerate the drug discovery and development process. By utilizing hPSC technology, models for many complex diseases can be established, which can take part in several stages of the drug discovery and development pipeline like lead discovery, in vitro studies, and post-clinical studies.

With the recognition of the value of hPSC-derived cell models, researchers have written many reviews on this topic. For example, Rowe and Daley [8] wrote a review on the use of iPSC-derived organoids in disease modeling, particularly emphasizing host-pathogen interactions and human–animal chimaeras. By analyzing a number of key central nervous system (CNS) disorders, Silva and Haggarty [9] presented phenotypic analysis of hPSC-derived models and their applications in drug screening. A more recent review by Garcia-Leon et al. [10] focused on drug screening in AD by using hiPSC-derived neurons and their related cell types. Contrary to focusing on a particular application or disease, we present the potential of hPSC-derived models during the whole process of drug discovery and development.

## 2. Overview of Current Drug Discovery and Development

The drug discovery pipeline involves several stages. The first is to use a certain target, a phenotypic screening method or a target-based screening method, to pick up one or more candidate molecules, which are also called lead compounds or leads. Lead compounds next go into medicinal chemistry programs for structure modification to enhance specificity, efficacy, and stability. During this stage, the effect of compounds is mainly tested in vitro using immortalized cells and/or primary cells. Then, the most effective compounds are directed into the stage of in vivo animal studies. Their toxicity, optimal dose, and delivery route need to be studied [11]. After being synthesized, a compound must be rigorously tested in preclinical studies to make sure that the drug under study is of sufficient efficacy, together with minimal side effects [12].

Lead discovery in target-based and phenotypic screening are usually based on immortalized cells, and evaluating efficacy and safety in preclinical trials are generally based on immortalized cells, primary cells, and animal models. Unfortunately, there are significant problems with each of these models. For example, there exist significant differences between cardiac electrophysiology in mice and humans, which can be indicated by heart rate—a human’s heart rate is generally around 70 beats per minute (bpm), whereas that of a mouse is about 600 bpm [13]. Compared to animal models, primary cells show human physiology and pathology in a more direct way, whereas the latter are difficult to obtain and maintain. For instance, enough human coronary endothelial cells, derived from human coronary arteries, are difficult to get for expansion because of the invasive procedure. Consequently, coronary endothelial cells usually have to be pooled, which makes it impossible to ascertain individual differences [14]. Therefore, a novel model is urgently needed to make drug development procedures more efficient with lower costs and higher accuracy.

As an alternative to animal models and primary cells, hPSC technology has caused a radical change in the field of drug discovery and clinical trials. Allowing for generating disease- and patient-specific functional somatic cells in a large scale, hPSC technology can avoid many problems that usually accompany animal and primary cell models.

Following success in preclinical studies, the selected compound enters the stage of clinical trials, which include phases I, II, and III. In phase I trials, the safety of the compound in the human body is tested, whereas in phases II and III, the efficacy and safety are tested in a larger number of patients. After accomplishing phase III, the candidate drug must seek permission from relevant regulatory agencies to enter the market [11].

Drug discovery and development is inherently risky. A major challenge in drug development is the high attrition rate before entering the market. According to research by DiMasi et al. [15], less than 11% of new pharmaceutical agents that entered clinical studies finally obtained marketing approval. The estimated clinical approval success rates for self-originated drugs varied noticeably by therapeutic class. The estimated approval success rates of CNS, cardiovascular, gastrointestinal/metabolism, and respiratory categories are relatively low at 8%, 9%, 9%, and 10%, respectively, whereas that of systemic anti-infectives is relatively high at 24% [16]. And the estimated average out-of-pocket cost per new drug is USD 403 million [15].

Another challenge in drug development is drug withdrawal from the market, which often results from a safety issue. For example, the COX-2 inhibitor rofecoxib was withdrawn because of severe vascular toxicity, causing huge financial losses to Merck. An analysis on drug withdrawal during 1990–2010 has found that 133 drugs were withdrawn because of safety reasons, including hepatotoxicity, cardiotoxicity, nephrotoxicity, and hypersensitivity [17]. Another larger study has identified 462 drugs that were withdrawn from the market during 1953–2013 because of adverse drug reactions, among which hepatotoxicity was the most common reason [18].

**Figure 1:**
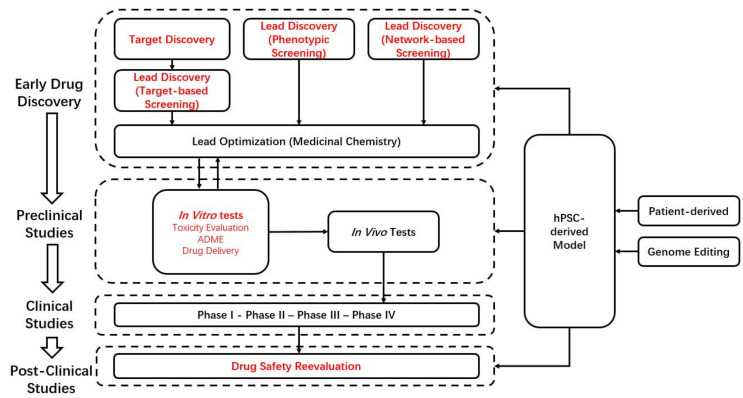
The workflow of drug discovery and development. Words in red font are stages that may involve hPSCs. ADME: absorption, distribution, metabolism, and excretion.

**Figure 2:**
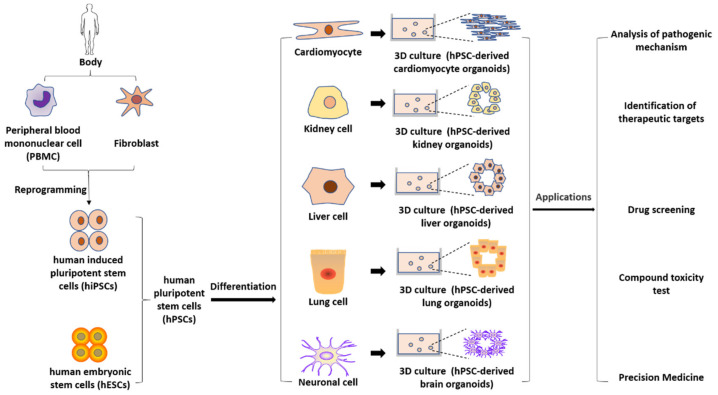
Types and possible applications of hPSC-derived models.

To reduce the loss from high attrition and withdrawal in drug development, the key point is to reduce failure in the stage of clinical trials. To achieve the goal, promoting efficiency and accuracy of drug screening and eliminating potential toxic and/or ineffective compounds before entering clinical trials are urgently needed. In the stages of drug discovery and preclinical studies, there are many processes in which hPSCs can play an active role (Figure 1). In the following sections, we reviewed the role of hPSC-derived models in disease modeling, target discovery, drug screening, and toxicity evaluation (Figure 2). Moreover, cells can be generated from multiple patients to carry out “clinical trials in a dish” [19,20].

## 3. hPSC-Derived Disease Models

Disease models are useful for probing the etiology and pathophysiology of a disease and are critical for efficient discovery and development of novel therapeutics. Disease models within the current pharmaceutical industry rely heavily on animals (like spontaneously hypertensive rats [21], the rat pilocarpine model of epilepsy [22], chromosome-engineered mouse models of Down syndrome [23]) or immortalized cell lines (like HBV genome-integrated stable cells, HepG2.2.15 as an HBV infection model [24], lymphoblastoid cell lines as a mitochondrial disease model [25], the neuroblastoma SH-SY5Y cell line as a PD model [26]), which are usually from human tumors. Compared with primary cells, tissue slices, or intact organs, these foresaid models manifest limited biological relevance, but the former also have shortcomings. For example, primary human hepatocytes (PHHs) are the current gold standard for in vitro liver cell culture models, but the limited supply and difficult logistics of PHHs make it difficult to use PHHs in a larger scale. In addition, PHHs are found to lose the hepatocellular phenotype progressively when cultured over extended periods. Moreover, the isolation procedure causes interindividual differences and cell alterations, leading to some result variations in experiments [27].

With similar features to primary cell types and higher accessibility [28,29] in combination with technologies such as 3D cell cultures, organoid technology, and microfabrication, hPSC-derived functional somatic cells are promising in offering more accurate disease models.

### 3.1. Models for the Study of Genetic Disorders

#### 3.1.1. Patient-Derived hPSC Models

The discovery of patient-derived hiPSCs has produced various types of cells that can be used as in vitro models for many tricky diseases for which there were previously none. Now there have been hiPSC models for many genetic disorders, including hepatological diseases, hematological and immunological diseases, and cardiac diseases.

Systemic lupus erythematosus (SLE) is a kind of chronic inflammatory autoimmune disease that may impact on any part of the body [30]. According to previous studies, SLE shows significant familial aggregation, and the incidence was consistently higher in homozygotes (24%) than in heterozygotes (2%) [31]. The pathogenesis involves dysfunction of both specific and nonspecific immunity [32], but the etiology remains unclear. For SLE, there is still no effective cure [33], and the current immunosuppressive therapy is of high cost, with an unignorable risk of side effects like various infections and cardiovascular disease [34]. Within the SLE patient group, the phenotype and stage of progression are various [35,36]; therefore, personalized treatment is needed for a better effect. By utilizing mononuclear cells of SLE patients, Li et al. [37] generated SLE patient-derived hiPSCs successfully, which offers a tool for exploration of the SLE disease mechanism and drug discovery.

Polycystic kidney disease (PKD), a common cause of end-stage renal disease, is a lethal single-gene disorder [38]. The difficulty to recapitulate kidney structures correctly in vitro is a hurdle in the further study of the PKD mechanism and drug development. Utilizing polydimethylsiloxane scaffolds and hiPSCs derived from a PKD patient with a PAX2 mutation, Benedetti et al. [39] generated ureteric bud-like 3D tubules, paving the way for studying the PKD mechanism and developing personalized medicine.

Neurofibromatosis type 1 (NF1), a tumor predisposition syndrome, is a common human genetic disorder affecting the nervous system. It is caused by mutations in the NF1 gene, leading to neurofibromas, which are peripheral nerve sheath tumors derived from Schwann cells. In order to study neurofibroma pathogenesis, Mo et al. [40] generated Schwannian lineage cells (SLCs) from a set of NF1 mutant patient-derived hiPSCs. They implanted hiPSC-derived SLCs into mouse sciatic nerve and discovered that NF1-null ones successfully formed authentic neurofibromas in the mouse nerve system, setting up a humanized neurofibroma model.

In summary, patient-derived hiPSC disease models are useful tools to discover effective drug treatments for diseases without well-understood mechanisms. These models can be further combined with phenotypic screening (see Section 4.2) to identify effective treatments. For disease mechanism and pharmacology studies, a drawback of patient-derived hiPSC disease models is the lack of true isogenic control, which makes data interpretation challenging.

#### 3.1.2. Genome-Edited hPSC Models

As an emerging technology, genome editing using engineered nucleases has been applied to modify target genes in virtually all types of cells. Nucleases used for targeted genome editing include transcription activator-like effector nucleases (TALENs) [41,42], zinc finger nucleases (ZFNs) [43], and clustered regularly interspaced short palindromic repeat (CRISPR)-associated nuclease Cas9 [44]. In 2012, Jinek et al. [45] first demonstrated that CRISPR-Cas9 could be used to specifically cleave target DNA and suggested its potential as a gene editing tool for eukaryotes. Within several months, CRISPR-Cas9 was proven to be capable to edit the human genome [46]. By now genome editing has been applied in hPSCs. We recently developed an improved CRISPR method based on Cas9 mRNA to target genes at highly condensed chromatin regions and achieved up to a 76% biallelic targeting efficiency in hPSCs [47]. Being able to be used for direct correction or insertion of interested genetic mutations in hPSCs, genome editing with programmable nucleases represents an effective tool for fundamental and preclinical research, including developing disease models [48,49].

Retinitis pigmentosa (RP) is a common type of hereditary retinal dystrophy, which involves primarily retinal cells and retinal pigment epithelial cells, ultimately resulting in blindness [50]. A severe form of X-linked retinitis pigmentosa (XLRP) is caused by RP2 mutations. In animal models of RP2 XLRP, the severe phenotype has failed to be recapitulated, so there is an urgent need for a proper model. Using the CRISPR/Cas9 technique, Lane et al. [51] knocked out RP2 gene in hiPSCs. Then, RP2-knockout (RP2-KO) hiPSCs, the non-edited isogenic control, and RP2 patient-derived hiPSCs were differentiated into 3D retinal organoids (ROs). Noticeably, rod photoreceptor cells in RP2 patient-derived and RP2-KO ROs exhibited a spike in cell death by day 150 of culture. Subsequently, the thickness of the organoid outer nuclear layer (ONL) was found to be less at day 180. To rescue the degeneration phenotype, adeno-associated virus was applied to mediate gene augmentation with human RP2 and was found to be effective in stopping ONL getting thinner as well as in recovering rhodopsin expression. In summary, this research set up a 3D model with hiPSC-derived ROs. Using the model, the phenotype of RP2 XLRP can be successfully recapitulated, and potential treatment can be tested.

Amyotrophic lateral sclerosis (ALS) is a neurodegenerative disorder that features progressive degeneration of motor neurons (MNs), which causes symptoms like muscle weakness, atrophy, and paralysis and leads to respiratory failure and death in the late stage [52]. Some familial ALS cases are caused by mutations in the superoxide dismutase 1 (SOD1) gene that encodes SOD1, an antioxidant enzyme-scavenging superoxide radical; but, the mechanisms remain unclear [53]. To understand the naturally occurring pathology of ALS and how the SOD1 mutation affects MNs, utilizing a CRISPR/Cas9 genome editing system on hiPSCs, Kim et al. [54] generated hiPSC-derived MNs harboring a knocked-in SOD1-G93A missense mutation. In this research, the wild-type MN cell line served as an isogenic control, while MNs generated from a patient-derived hiPSC line harboring another form of SOD1 mutation served as a positive control. In the cell bodies of MNs with either a G93A or A4V mutation, misfolded and aggregated forms of SOD1 were accumulated, including axons. Additionally, they showed distinctive axonal pathologies including larger and shorter axons and less branch points. In addition, structural and molecular abnormalities were identified in presynaptic and postsynaptic structures. Furthermore, aberrant neurotransmission was identified in mutant MNs. All these disease phenotypes relevant to ALS indicate that genome-edited hiPSCs utilizing CRISPR/Cas9 technology and MNs derived from them, together with their proper control cells, are important to the modeling of ALS and the study of disease mechanisms in human ALS.

KCNQ1 and KCNH2, which encode potassium channels, are important genes leading to long QT (the time from the start of the Q wave to the end of T wave on an electrocardiogram) syndrome (LQTS) [55]. Wang et al. [56] inserted the ion channel genes KCNQ1 and KCNH2 into the genome of hPSCs by the ZFN technique. The edited hiPSC-derived cardiomyocytes (hiPSC-CMs) showed characteristic LQTs and a significantly longer action potential duration (APD) than unedited cardiomyocytes. At the same time, APD was significantly shortened when hiPSC-CM was treated with nifedipine (an L-type calcium channel blocker) or pinanardil (a KATP channel opener). The results showed that the gene-edited hiPSC-CMs successfully constructed the LQTS model in vitro and provided materials for studying the mechanism of LQTS and drug screening.

In summary, genome editing technology can generate true isogenic controls with identical genetic background except the gene of interest, which overcomes the drawback of patient-derived hPSC models. However, the knowledge of disease-causing genetic information is a prerequisite for genome editing.

### 3.2. Models for the Study of Acquired Diseases

Neurodegenerative diseases are a kind of serious acquired disease, which include AD, PD, multiple sclerosis, etc. [57]. They feature a chronic and progressive deterioration of neuronal function, leading to cognitive impairment, impaired motor function, memory loss, and sensory and emotional changes in patients. While studying animal models of neurodegenerative diseases, Dawson et al. [58] proposed that many neurodegenerative diseases share the same pathological manifestations, including abnormal accumulation of toxic aggregates [59], oxidative stress, mitochondrial dysfunction [60], defective axon transport [61], and chronic inflammation [62], which eventually lead to neurodegenerative diseases. Because of the complex pathogenesis of neurodegenerative diseases, currently, there is no specific drug for neurodegenerative diseases in clinical practice, only symptomatic drugs that can relieve the symptoms. Modeling of neurodegenerative diseases is also a challenge. While animal models of neurodegenerative diseases have emerged [58], they require the use of many animals and a large amount of time, and the differences between species also make animal models have certain limitations. With the development of science and technology, hPSCs have brought hope to people. Somatic cells were extracted from patients with neurodegenerative diseases and reprogramed to hiPSCs by pluripotent factors. Different differentiation factors then induce differentiation into specific nerve cell types, including dopaminergic neurons, cholinergic neurons, astrocytes [63], and so on. Using these nerve cells to construct models of neurodegenerative diseases can greatly solve the problems existing in animal models.

The histopathological features of AD are the aggregation of β-amyloid peptide (Aβ) and the protein tau tangled together to form plaques [64]. Currently, rodent models of AD are widely used in clinical practice, but there are some differences between rodent models and human models, and a human model is the gold standard for studying AD. Therefore, the construction of a human AD model has become the research direction of many scholars. The development of hiPSC technology has made it possible to model AD in vitro. By using CRISPR-FokI technology, García-León et al. [65] created three mutations (N279K, P301L, and E10 + 16) in the 10th exon and adjacent region of the MAPT gene, which encodes tau. These mutations caused tau aggregation in hiPSC-derived neurons and glial cells accompanied with a series of neurodegenerative changes, such as inflammation, oxidative stress, and electrophysiological alterations. Using this model, the authors were able to study AD in vitro. hiPSC-based systems and genome editing tools can help to figure out the mechanism of neurodegenerative diseases and promote the research of AD and other neurodegenerative diseases.

PD is caused by the loss of dopaminergic neurons in the substantia nigra striatum system. Transplantation of dopaminergic neurons has become one of the approaches to treat PD. Clinically, drug therapy is mainly used because of its low cost, high patient compliance, and its being non-invasive. The emergence of hPSCs has made cell therapy become a research hotspot. Cell transplantation therapy has been proven to be safe and effective. Dopaminergic neurons derived from autologous hiPSCs were transplanted into non-human primates. They survived for two years without immunosuppression and the animal’s nervous system recovered [66]. In 2016, International Stem Cell Corporation initiated the first approved clinical trial of hiPSCs to treat patients with PD in Melbourne, Australia [67]. In the future, the combination of cell therapy and drug therapy may become one of the important methods to treat PD.

Currently, many cell types derived from hiPSCs have molecular features like fetal cells based on transcriptomic, structural, or functional studies, and the in vitro differentiation of hiPSCs can faithfully mimic in vivo embryo and fetal development [68,69,70], which makes this model particularly useful in studying disease development at the prenatal stage. However, for those acquired diseases that develop with age, hiPSC-derived cell models may have less potential. Attempts have been made to improve in vitro maturation. For example, Burke et al. [71] co-cultured hiPSC-derived neuronal cells with astrocytes and obtained 48% of transcripts representing mature cortical neurons. Kolanowski et al. [72] cultured hiPSC-derived cardiomyocytes in a microfluidic device that provided pulsatile hemodynamic signals to the cells. This method increased the alignment and mitochondrial functions of the cells.

## 4. hPSC-Derived Models in Drug Discovery and Development

### 4.1. Target Discovery

A drug target can be defined as “a molecule in the body, usually a protein that is intrinsically associated with a particular disease process and that could be addressed by a drug to produce a desired therapeutic effect” [73]. Discovering a proper therapeutic target is the basis of the classic drug development pipeline. Models derived from hPSCs, amenable to scale up and like primary cell types, are of potential use in discovering therapeutic targets in fundamental research.

A major cause of mitochondrial diseases is mutations in mitochondrial DNA (mtDNA). The cells with disease-related mutations in mtDNA present a series of phenotypes like reduced respiration and increased lactification. hiPSCs derived from patients with mitochondrial disease, with high proportions of mutated mtDNA, display maturation defects. Kobayashi et al. [74] discovered that tryptolinamide (TLAM), a small-molecule compound, activates the function of mitochondria in hybrid cells generated from anucleate cytoplasm of patient-derived cells and in fibroblasts differentiated from patient-derived hiPSCs. They found that TLAM is an inhibitor of phosphofructokinase-1 (PFK1). Increasing the level of AMP-activated protein kinase-mediated acetyl-CoA carboxylase phosphorylation, TLAM promotes oxidative phosphorylation and shifts energy metabolism from glycolysis to mitochondrial respiration. Moreover, TLAM rescues the phenotype of patient-derived hiPSCs, suggesting that PFK1 is of potential to become a disease-modifying target for mitochondrial diseases.

N-methyl-D-ionic glutamate receptors (NMDARs) are neuron-expressing ionotropic glutamate receptors. Studies in various animal models have found that Src family kinases are involved in brain development and activities by regulating the function of NMDARs. Data from human neurons are scarce. By using hiPSC-derived neurons, Zhang et al. [75] found that Fyn, a member of Src family kinases, promoted the function of GluN2B subunit-containing NMDARs, indicating that Fyn could be a drug target to regulate neuron functions.

In summary, hPSC-derived models help to identify therapeutic targets, paving the way for subsequent research for developing effective therapies.

### 4.2. Phenotypic Screening

Phenotypic screening is a strategy of lead discovery, which is based on measurable phenotypic endpoints from cells or organisms without having prior knowledge of the drug target [76]. Phenotypic screening offers an unbiased approach to “chemically interrogate the proteome in its pathophysiologically-relevant environment” and promote opportunities to uncover the true disease mechanisms and to identify potential therapeutic drugs [77]. In recent years, phenotypic screening is becoming popular for identifying disease-modifying bioactive compounds [78], particularly for diseases where critical therapeutic targets have been hard to identify in other ways [79]. Thus, phenotypic screening may contribute to reducing high attrition rates in drug development, especially the failures in phase II, which usually result from insufficient efficacy. hPSC models are promising in providing platforms for phenotypic screening to identify candidate drugs for many intractable diseases.

Tuberculosis (TB), caused by *Mycobacterium tuberculosis* (Mtb), is a major infectious disease over the world [80]. Standard therapy for drug-sensitive TB involves core antibiotics including isoniazid, rifampicin, pyrazinamide, and ethambutol. However, TB treatment is faced with the challenge of multidrug-resistance (MDR) and extensive drug-resistance (XDR), so there arises an urgent need for novel anti-TB drugs. The entire genome sequence of Mtb was described in 1998, which facilitated identifying new drug targets and developing drug screening based on new targets [81]. However, not many targets have been identified so far, and for quite a few vital ones, there are no specific inhibitors of clinical value. There have been no clinically effective anti-TB drugs discovered adopting target-based strategy [82]. To overcome the MDR problem and to move beyond the strategy based on classic targets, phenotypic screening has been an acceptable alternative.

Han et al. [83] developed a modified protocol for using hESCs to generate homogeneous populations of macrophage-like cells (iMACs), which showed similar transcriptomic profiles and had characteristic immunological features of classical macrophages. Moreover, iMAC production could be scaled up. Using iMACs infected with Mtb H37Rv-GFP9 (a mycobacterial laboratory strain, modified to express GFP), they performed a high-throughput phenotypic screening against intracellular Mtb, involving a library of 3716 compounds. In the primary screening, there were 120 hits identified, then a secondary screening was performed by dose-intracellular and -extracellular Mtb assays. Finally, a new anti-Mtb compound named 10-DEBC was identified, which showed activity against drug-resistant Mtb strains as well.

For ALS, there has been no effective treatment or common therapeutic target. In a study conducted by Imamura et al. [84], motor neurons differentiated from ALS patient-derived hiPSCs were used for phenotypic screening. In the screening, survival of motor neurons differentiated from ALS patient-derived hiPSCs was used as an endpoint. They screened existing drugs and found that Src/c-Abl kinases inhibitors promoted autophagy and rescued ALS motor neurons from degeneration. Bosutinib, one of Src/c-Abl kinases’ inhibitors, was effective for increasing the survival of ALS patient-derived motor neurons.

### 4.3. Network-Based Screening

Traditional small-molecule screening approaches aiming at identifying therapeutic candidates generally search for molecules involving merely one to several outputs, which limits the discovery of drugs with actual therapeutic effects. In 2007, A.L. Hopkins pioneered the concept of “network pharmacology” [85]. The concept is based on the theories of systems biology, genomics, proteomics, polypharmacology, etc. and uses technologies such as histology, high-throughput screening, network visualization, and network analysis to reveal the complex biological network relationships among drugs, genes, targets, and diseases, based on which the pharmacological mechanisms of drugs are analyzed and predicted [86]. Based on network pharmacology, a network-based screening strategy provides a holistic perspective of disease mechanisms, offering an unbiased approach to assess a drug’s therapeutic effect and to identify disease-modifying drugs. Recently, hPSC-derived models have been used in the construction of gene networks in network-based screening.

AD is a common cause of elderly dementia, affecting over 40 million patients all around the world [87]. Symptoms of AD include loss of memory and decline in cognitive functions, resulting from neuronal impairment and death, which is accompanied by brain inflammation [88]. AD is a multifactorial disease, which involves several regulatory processes, like lipid metabolism, vesicle trafficking, and endocytosis [89,90]. Based on different onset mechanisms, several subtypes of AD have been defined [91].

Considering the diversity of risk factors and onset mechanisms, it is difficult to discover a disease-modifying target for all AD patients if the screening strategy focuses on a single pathway. Thus, there arises a need for an approach that takes all existing genetic factors and relevant regulatory pathways into consideration to search for an optimal therapeutic target [92].

To develop effective drugs for AD, Park et al. [93] set up a network-based drug-screening platform combining mathematical modeling and hiPSC technology. They constructed a mathematical AD signaling model integrating relevant pathways validated with iCOs, which are cerebral organoids differentiated from hiPSCs (including patient-derived and CRISPR-Cas9-edited hiPSCs) and built a high-content screening system using 1300 organoids from 11 participants, providing a platform for drug assessment and precision medicine.

By combining machine-learning and hiPSC technologies, Theodoris et al. [94] developed an approach to search therapeutic candidates for aortic valve (AV) disease. It is reported that heterozygous loss-of-function NOTCH1 (N1, a transmembrane receptor that functions as a transcriptional regulator) mutations cause AV stenosis and calcific AV disease [95]. Utilizing machine learning, they drew a map of gene networks dysregulated by N1 haploinsufficiency with hiPSC-derived endothelial cells and identified an efficacious therapeutic candidate that can correct the network dysregulation, XCT790. Moreover, the effectiveness of XCT790 was generalized to primary AV cells from over 20 AV patients and a mouse model.

### 4.4. Models for the Study of Disease Mechanisms

Muscular dystrophies (MD) comprise a group of hereditary and progressive muscle diseases that result from a number of different gene mutations, of which terminal pathology often represents muscle necrosis and replacement by fibrotic or fatty tissues. Lacking appropriate models, the studies of MDs are limited. Being able to generate specific cell types like skeletal muscle fibers and cardiomyocytes affected in a certain type of MDs, hiPSCs offer a useful model for studying the disease mechanisms of MDs [96].

LGMD2I, a type of MD, is caused by fukutin-related protein (FKRP) gene mutation [97]. More than half of LGMD2I patients had cardiac involvement like progressive dilated cardiomyopathy [97,98,99]. Because live human cardiac cells are difficult to access and animal models failed to demonstrate cardiomyopathy, the detailed molecular or electrophysiological mechanism has not yet been defined [100]. A study conducted by El-Battrawy et al. has shed light on the pathogenesis [101]. They generated hiPSC-derived cardiomyocytes from an LGMD2I patient with dilated cardiomyopathy and found that Na^+^, Ca^2+^, and K^+^ channel dysfunction resulted in a reduction in amplitude and upstroke velocity of action potentials as well as decreased Ca^2+^ release. The former may impair the conduction of heart excitation and the rhythm, whereas the latter may reduce the contraction force of cardiomyocytes and lead to dilated cardiomyopathy. This patient-derived hiPSC cardiomyocytes model is promising in mechanistic studies of LGMD2I.

AMD, taking up over 50% of newly certified vision impairment in England and Wales, is a leading cause of blindness [102]. AMD is a progressive disease involving multiple factors, including environment, metabolism, immunity, and genetics [103]. For AMD, there are two advanced forms, “wet” and “dry”. For the former, anti-vascular endothelial growth factor agents have been widely used [104,105], whereas, for the latter, there exists no effective treatment. To develop a disease-modifying therapy for dry AMD, further studies for the disease mechanisms are needed. Some evidence shows that an association exists between AMD and a dysfunctional autophagy-lysosome pathway [106,107,108], but the actual role autophagy plays in AMD pathophysiology remains unclear because of a lack of a satisfactory human in vitro AMD model. Edvinas et al. [109] used retinal pigment epithelium (RPE) cells generated from Y402H (complement factor H (CFH) polymorphism Y402H)-AMD-patient-derived hiPSCs to set up an in vitro model and discovered that in Y402H-AMD-patient-specific RPE cells, the significantly increased C3 turnover led to autophagy dysfunction by increasing deposition of the terminal attack complex C5b-9 at the lysosomes, which resulted in lysosomal overburden and malfunction. Moreover, they found that by inhibiting C3 turnover with the compstatin analogue Cp40, all cellular disease phenotypes were reversed. This research shows a new link between the complement system and the autophagy–lysosome axis, contributing to revealing the disease mechanisms of AMD.

In summary, utilizing hPSC technology, we can develop disease models that previously could not be built, which helps to figure out the mechanisms of many intractable diseases.

### 4.5. Models for Toxicology

The safety test and toxicology test of a drug are an important part of the preclinical research. Drugs must be tested for safety and toxicity before they are marketed. In general, the commonly used parameters to indicate drug toxicity are the half lethal dose (LD50) or the half lethal concentration (LC50), the half effective dose (ED50), the minimum lethal dose (MLD) or minimum lethal concentration (MLC), the maximum tolerable dose (MTD) or maximum tolerable concentration (MLC), the therapeutic index (TI), the minimum effective dose (MED), the minimum toxic dose (MTD), etc. LD50 is the most important parameter of the safety test and the toxicity test. LD50 is the dose needed for a drug to cause 50% death in a group of laboratory animals, and it is obtained by experiments [110]. TI is the ratio of the LD50 to the ED50 [111]. The greater the value of TI, the smaller the ED50 and the larger the LD50 of the drug, indicating that the drug is safer. The therapeutic window is the concentration of the drug between the MED and the MTD. The drug is effective when the concentration of the drug is within the therapeutic window.

At present, there are many models used to determine the safety of drugs, including rodent models, mammalian models, small organism models, cell models, and organoid model. A common rodent model is the mouse model [112]. However, because of species differences between humans and rodent animals, the metabolic enzymes are different. The disposition of a drug between humans and animals is different [113], so the results are going to be different. Mammalian models include the pig model [114], the rabbit model [115], and so on. Mammalian models have been considered the gold standard for testing drug toxicity because mammals share the same developmental pathway as humans, and most of their organs, metabolic enzymes, and metabolic pathways are similar to humans. However, the high cost of animal models and the ethical and moral problems cannot be ignored. Small organism models include the zebrafish model [116] and the caenorhabditis elegans model [117]. Using small biological models for drug toxicity testing can obtain the reproductive, endocrine, and nervous system data of a complete individual, and the cost of small biological models is much lower than that of mammalian animal models, but it still cannot completely replace mammalian animal models. Common cell models include the liver cell model, the nerve cell model, the myocardial cell model, and so on. Although these models can reflect drug toxicity, they are sometimes inaccurate. For example, when using the liver cell model to evaluate drug-induced liver injury, it is not enough to use only liver parenchymal cells. The damage of liver non-parenchymal cells can also lead to liver injury. The abnormal function of Kupffer cells will lead to abnormal liver microcirculation and affect the function of liver cells. Abnormal hepatic stellate cells can lead to liver fibrosis. Through the above analysis, we can learn that each model has its own advantages and disadvantages, and researchers can choose a suitable model according to their own needs. With the development of hPSC, a low-cost, simple, convenient, and exact model has emerged. Based on cells differentiated from hPSCs, we can construct organoid models that have homology with the donor without species differences and can fully simulate the process of drug metabolism in the organ to evaluate the effects of drugs on a variety of cells, meaning we are no longer limited to one kind of cell. Organoid models can be made in a petri dish, require less starting material, and cost less. Based on these advantages, organoid models have soon become a new model for studying drug toxicity in vitro. At present, the organ-like model has been established, including brain organoids [118,119], hPSC-derived blood–brain barrier models [120], hPSC-derived cardiomyocyte organoids [121], liver organoids [122], and kidney organoids [123,124].

Sirenko et al. [125] used the hiPSC-derived model of liver cells to analyze and assess the hepatotoxicity of 240 compounds by observing the cell vitality, the nucleus shape, the average cell area and consolidation, the mitochondrial membrane potential, the accumulation of phospholipids, cytoskeleton integrity, and apoptosis, which facilitates the safety assessment of drugs and chemicals. Mun et al. [126] gradually differentiated hiPSCs into mature hepatocytes (MH) in vitro. About 22 days after differentiation, MHs began to appear as 3D spherical structures and showed a 3D morphology similar to liver parenchymal cells. When MHs embedded in Matrigel were cultured in a liver medium, liver organoids were significantly enlarged and showed the ability of liver to self-renew. It was found that the liver organoids could still maintain normal nuclear morphology after three months of culture in vitro, had glycolysis activity, and could perform the tricarboxylic acid cycle and oxidative phosphorylation. At the same time, liver organoids expressed sufficient levels of products that are expressed by mature hepatocytes, such as ALB, TTR, duct-labeled CK19, and those in the basic cytochrome CYP family (3A4, 1A2, 2A6, and 2E1). In predicting toxicological outcomes using troglitazone and acetaminophen, 3D liver organoids have advantages over two-dimensional (2D) models. Thus, the liver organoids have natural drug metabolic activity and toxicity sensitivity, which can be used for toxicity prediction, drug screening, and disease modeling.

Sirenko et al. [127] used hiPSC-derived models to evaluate the cardiotoxicity of 69 representative environmental chemicals in vitro and found that environmental pollutants altered the function of cardiomyocytes in vitro at high exposure levels, and similar chemicals had similar effects on cardiomyocytes in vitro. There have been many reports of using hiPSC-derived models to determine the cardiotoxicity of compounds or drugs [128,129,130]. Ni et al. [131] constructed an in vitro cardiovascular cell model using cardiomyocytes (hPSC-CMS) and endothelial cells (hPSC-ECs) induced by homologous hPSCs. The ratio of myocardial troponin T (TNNT2)-positive cardiomyocytes in this model was more than 90%, and the cardiomyocytes had normal myofilament structures and electrophysiological characteristics. CD31 and CD144 double positive hiPSC-EC accounted for more than 90%. These results demonstrate that cardiovascular cell models were established in vitro. The new safe lipid-lowering drug alirocumab, the dose-dependent cardiotoxic drug atorvastatin, and the cardiotoxic drug doxorubicin were used to verify the model, and the results were consistent with the theory. It has been proved that the safety and toxicity of lipid-lowering drugs can be evaluated by this model in vitro and can promote the research and development of lipid-lowering drugs.

With advances in cell biology, other researchers have used hiPSC-derived models to measure the nephrotoxicity [132], neurotoxicity [133], muscle toxicity [134], and other toxicities of drugs or other compounds. It is important to take into account the differences between developing and mature nerve cells, as well as the role of the blood–brain barrier, when testing drugs for neurotoxicity. The models of neurons that have been differentiated from hiPSCs include the dopaminergic neuron model [135], the cholinergic neuron model [136], the astrocyte model [137], and models of the blood–brain barrier [138]. Glutamate is an excitatory neurotransmitter, and one of its receptors is NMDAR, which was mentioned in Section 4.1. NMDAR is important for studying the effects of chemicals on neurotransmitters, but NMDAR has not been found in conventional nerve cell lines or in most stem-cell-derived neurons. Klima et al. [139] used PSC to cultivate a mixed cortical culture (MCC). Immunocytochemistry and gene expression profiles showed that MCC contained a variety of neurotransmitter receptors including NMDAR. Verified by neurotransmitter agonists and antagonists, MCC based on PSC pioneered a cellular model of the effects of chemicals on neurotransmitter receptors. Mitochondrial dysfunction can seriously affect the normal functioning of the nervous system. For ethical reasons, the current models for studying mitochondrial dysfunction are postmortem brain specimens, animal models, and 2D nervous systems [140]. However, these models do not fully mimic the complex human nervous system. Lancaster et al. [141] have developed a brain organoid model with multiple types of brain cells and 3D structures, which has become a powerful tool for modeling and evaluating mitochondrial disorders. However, this model uses hiPSCs derived from human skin fibroblasts, and the acquisition process is slow and susceptible to external environmental factors. Human skin fibroblasts are often exposed to ultraviolet radiation, which will cause DNA changes. If hiPSCs induced by the mutated fibroblasts are established using an in vitro model, the status of the patient cannot be accurately reflected. So, there are some defects in this model. Duong et al. [142] used peripheral blood mononuclear cells (PBMCs)-derived hiPSCs to construct a human brain organoid model. Because PBMCs are less affected by the environment, they mutate less than skin fibroblasts; thus, PBMCs-derived hiPSCs can more accurately reflect the patient’s status. Brain organoid models derived from PBMCs have been used as in vitro model of mitochondrial disorders.

In summary, toxicity tests of hepatotoxicity, cardiotoxicity, neurotoxicity, musculotoxicity, and nephrotoxicity using hiPSC-derived models in vitro can predict the metabolism and dose range of drugs, can understand the potential toxicity of drugs, and can greatly improve the safety of drugs. It is precisely because of the numerous advantages of hiPSC-derived models that they are believed to replace the existing animal models in drug safety and toxicity testing in the near future.

### 4.6. Models for Precision Medicine

Jameson et al. [143] defines precision medicine as “treatments targeted to the needs of individual patients on the basis of genetic, biomarker, phenotypic, or psychosocial characteristics that distinguish a given patient from other patients with similar clinical presentations.” However, König et al. [144] believe that precision medicine is a constantly changing process, constantly collecting changes in variables and timely feedback to the in-depth study stage, assessing the patient’s status, and then performing individualized treatment. To this end, what can hPSC-derived models contribute to precision medicine?

Precision medicine includes three aspects. The first one is big data analysis, which is the foundation of precision medicine and can provide multi-dimensional data. Through the collection and analysis of population data, we can analyze the real source of the disease and find the most appropriate treatment plan. The second aspect is accurate diagnosis, which can provide more detailed and accurate patient data and can help the big data system to better judge the patient’s condition. Genetic testing [145,146] has demonstrated its remarkable contribution to precision medicine. The third aspect is precision treatment, which can reduce the adverse reactions, implement more effective treatment, and even cure the difficult diseases that could not be solved in the past.

With the continuous progress of hPSC technology, hPSCs are playing an increasingly important role in precision medicine. Firstly, hPSCs can be used to construct patient-specific disease models in vitro, from which we can gain a deeper understanding of the pathogenesis of patients, and these patient-specific disease cells can be used for drug screening to achieve the most effective treatment. Tai et al. [147] have successfully reprogrammed human skin fibroblasts into hiPSCs using non-integrated Sendai virus. If the skin cell comes from a patient, the hiPSC shares the same genes as the patient and could help mimic the patient’s disease [148].

Secondly, the most effective method for drug screening based on the disease model established by hPSCs is to treat according to the target. The progress of hPSC technology has made it possible to create a disease model for each patient. We can understand the progression of the disease and select the drug that is most suitable for that patient. Strikoudis et al. [149] used CRISPR/Cas9 to introduce a frameshift mutation in the Hermansky–Pudlak syndrome (HPS) gene and found that the edited hESC-derived lung organoids showed fibrosis changes and increased interleukin-11 (IL-11) expression. They also found that IL-11 induced fibrosis in wild-type lung organoids, whereas IL-11-deprived HPS lung organoids did not develop fibrosis. This suggests that IL-11 is a therapeutic target of idiopathic pulmonary fibrosis (IPF). Drugs that antagonize IL-11 may be used to target IPF to minimize adverse reactions and maximize therapeutic effects.

At the moment, doctors usually diagnose cancer patients clinically through pathological tissue sections, and cancer patients are treated with chemotherapy or radiation, which is very harmful to patients. It is believed that hPSC-derived models will provide important help in disease diagnosis in the future. We can take skin cells from patients and reprogram them into hiPSCs, and then differentiate them into organoids. The organoid models can not only provide us with the pathogenesis of cancer but also with therapeutic targets, according to which we can effectively treat patients, greatly saving their lives and alleviating their suffering.

Similarly, other researchers have applied hiPSCs to the precision treatment of human liver diseases [150] and central nervous system diseases [9]. hPSC-derived models play a significant role in precision medicine and run through the whole process of precision medicine. It can provide doctors with the pathogenesis of a disease, and we can use genetic testing to find possible therapeutic targets for a disease; then, through drug screening based on this target, we can find the most suitable drug for the patient. hPSC has brought great convenience to doctors and patients. This may be one of the most important treatments for genetic diseases and cancer in the future. Organoid models in hPSC-derived models may make important contributions to organ transplantation. This has also become a hot research direction recently.

### 4.7. Clinical Trial in a Dish

In the past, preclinical studies of drugs have been conducted in animals, and clinical trials have been conducted in humans. The emergence of hiPSCs has opened new avenues for preclinical research and clinical trials to investigate drug safety and toxicity at the human cellular level. This method is called clinical trial in a dish (CTID) [151]. CTID can be used to test a medical treatment for the safety or efficacy on a specific patient’s cells. CTID can be used in population-specific drug development to predict not only how effective a drug will be but also how adverse reactions to the drug will be before actual clinical trials. At the same time, after CTID, researchers will choose safer drugs to enter clinical development, thus reducing drug research expenditures.

Enlarged ventricles and dysfunction are characteristic of dilated cardiomyopathy (DCM) [152]. Severe cases can result in heart failure and are kept alive by a heart transplant. Familial DCM involves more than 60 genes, the most common of which is the genetic variation that encodes the nuclear envelope proteins Lamin A/C (LMNA) [153]. LMNA mutations that cause lipodystrophy or progeria show endothelial cell (EC)-dependent vascular dysfunction leading to premature atherosclerosis [154]. In a study [155] of the mechanism by which lovastatin improves LMNA-associated dilated cardiomyopathy in vitro, Sayed et al. found that patients with LMNA-associated DCM showed clinical endothelial dysfunction and reduced function of hiPSC-derived ECs (hiPSC-ECs) derived from these patients, such as angiogenesis disorder and nitric oxide production disorder. At the same time, the restoration of hiPSC-EC function with LMNA mutation was corrected by gene editing technology. Finally, it was found that the expression of Kruppel like factor 2 of hiPSC-EC in patients with LMNA mutation was suppressed, leading to reduced cell function. Thus, the reactive hyperemia index of LMNA-related DCM patients treated with lovastatin increased, indicating that the clinical endothelial dysfunction was improved. In addition, hiPSC-EC from patients with LMNA mutations treated with lovastatin were co-cultured with hiPSC-derived cardiomyocytes (hiPSC-CM) from patients with DCM who showed improved cardiomyocyte function. At the same time, some researchers [20] used hiPSCs for CTID, combined with genomic analysis, to identify patients vulnerable to cardiotoxicity induced by specific drugs, thus enhancing drug safety in these patients and reducing the incidence of adverse reactions. In addition, biomarkers for the treatment of disease can be found when CTID is performed with hiPSCs. Han et al. [156] found that the levels of circular RNA E3 ubiquitin-protein ligase (CircITCH) in both hiPSC-derived cardiomyocytes and patients who suffered from doxorubicin-induced cardiomyopathy decreased. However, cardiotoxicity was successfully prevented in mice induced by doxorubicin-induced toxicity after CircITCH overexpression using an adeno-associated virus serotype 9 vector. These results indicate that CircITCH is an important biomarker of doxorubicin-induced cardiomyopathy, and, based on this, drugs targeting doxorubicin-induced cardiomyopathy can be designed. In addition, the use of patient-specific hiPSCs for CTID can also identify at-risk populations, simulate clinical trials of toxic drugs [157], and identify the toxicity of drugs [158].

There have been several studies exploring the usefulness of hiPSC-derived cardiomyocytes in predicting drug-induced cardiotoxicity. The results vary. Stillitano, et al. [159] selected extreme patients who were either supersensitive or insensitive to drug-induced long QT and generated subject-specific hiPSC-derived cardiomyocytes. The authors found a good correlation between the response of patients with the response of their corresponding in-vitro-cultured hiPSC-derived cardiomyocytes to a cardiotoxic drug sotalol. In another study, Shinozawa, et al. [160] took a similar approach and found a positive correlation between the clinical QT intervals and the field potential duration prolongation values generated from cell assays at relevant concentrations of a QT-prolonging drug moxifloxacin. In contrast, a more recent study by Blinova et al. [161] failed to find a correlation between in vivo and in vitro data. The authors discussed that the possible reasons could be immaturity of hiPSC-derived cardiomyocytes, variation in stem cell differentiation, loss of epigenetic signatures, variation in cohort, and variation in the tested drug concentration range. Therefore, improving maturation and standardizing differentiation protocols are urgently needed.

To sum up, hiPSCs have been used by many researchers to carry out CTID, and some achievements have been made. Compared with traditional preclinical and clinical studies, CTID can save money and time and prevent serious adverse reactions. Although hiPSCs have not been used for a long time in CTID, we believe that hiPSCs have potential in drug discovery, prediction of adverse reactions, diagnosis, and treatment of diseases in the future once technical problems have been overcome.

### 4.8. Post-Clinical Studies

Post-clinical study is the stage of applied research after a new drug is marketed. Its purpose is to study the efficacy and adverse reactions of the drug in the case of widespread use, to evaluate the benefits and risks of the drug when used in general or special populations, and to improve its dosage. Here, we propose that, by establishing hiPSCs from patients who have used the drug of interest, we will be able to correlate the data of clinical drug use with hiPSC-derived in vitro models and then dissect the mechanisms of drug effect and toxicology. The results will be useful information to guide the rational use of drugs and the re-evaluation of drug safety.

## 5. Perspective

Owing to the capability of unlimited self-renewal and differentiating into any human somatic cells, hPSCs have great potential to build in vitro models for drug discovery and development. hPSC-derived in vitro models, either directly from patients or modified by genome editing technology, can improve the cost-effectiveness and the success rate of drug discovery and development. These models play an active role in phenotypic screening, target-based screening, network-based screening, and post-clinical studies for disease mechanism, drug development, rational drug use, and drug safety re-evaluation.

In vitro models for drug testing should be robust, reproducible, scalable, and cost-effective. Scale-up culture or progenitor cell expansion enable the production of large numbers of cells of interest. For hPSC-derived hepatocytes, researchers have developed different ways to increase the yield [126,162,163,164,165,166]. Reproducibility remains as an issue to be addressed. Poor reproducibility is due to unstandardized differentiation protocols and incomplete cell characterization. A recent review by Volpato and Webber nicely summarized the reasons and possible solutions to this issue [167]. The differentiation of hPSCs is a complex and time-consuming process, requiring different growth factors and extracellular matrix components, which dramatically increase the cost of the experiments. One of the strategies to make it more cost-effective and to allow pharma industries to adopt this model is the use of small molecules to replace growth factors. Immaturity of hPSC-derived cells is another problem that researchers currently tackle. Ethical issues related particularly to brain organoids has drawn attention in this field. Developing mature brain organoids for drug discovery and development may face a dilemma. On the one hand, we need mature brain organoids to model diseases and to test drug candidates. On the other hand, mature brain organoids with consciousness or cognition should not be developed and used in drug testing. This issue is waiting for updated regulation from stem cell societies and authorities [168]. Hopefully with the progress of related techniques, such as organoid technology, genome editing, and direct differentiation, hPSC-derived models will bring more benefits to the health of human beings.

## Data Availability

Not applicable.
